# Variation in alpha radioactivity of plants with the use of different fertilizers and radon measurement in fertilized soil samples

**DOI:** 10.1186/2052-336X-12-70

**Published:** 2014-04-22

**Authors:** Pooja Chauhan, Rishi Pal Chauhan

**Affiliations:** 1 Department of Physics, National Institute of Technology, Kurukshetra-136119, India

**Keywords:** Radioactivity, Soil, Fertilizer, Plants, Alpha track density, Radon concentration

## Abstract

**Background:**

People are exposed to ionizing radin from the radionuclides that are present in different types of natural sources, of which phosphate fertilizer is one of the most important sources. Fertilizers are commonly used in agricultural field worldwide to enhance the crop yield.

**Materials and methods:**

In the present investigation, a control study was carried out on the lady’s finger plants grown in earthen pots. To observe the effect of fertilizers their equal amounts were added to the soil just before the plantation. The alpha track densities were measured using solid state nuclear track detectors (SSNTDs), a sensitive detector for alpha particles.

**Results:**

The measured alpha track densities (T cm^−2^d^−1^) in lady’s finger plants on top and bottom face of leaves after 30, 50 and 70 days of plantation varied from 49 ± 11 to 206 ± 2.6, 49 ± 16 to 248 ± 16 and 57 ± 8.5 to 265 ± 32 respectively in various leaf samples.

**Conclusions:**

The alpha track densities were found to vary with nature of fertilizers added to the soil and an increase was also observed with time. The alpha track densities were also measured in soil samples mixed with different fertilizers. The radon exhalation rates in various soil samples and soil to plant transfer factor (TF) of alpha tracks were also calculated.

## Introduction

Natural ionizing radiation is emitted as a result of spontaneous nuclear transformations of unstable radionuclides naturally occurring in the earth’s crust (i.e. terrestrial origin) as well as those coming from outer space into the atmosphere (i.e. extraterrestrial origin) [1]. Natural radioactive materials under certain conditions can reach hazardous radiological levels. So, it is felt necessary to study the natural radioactivity in soil to assess the dose to the population in order to know the health risks and to have a baseline for future changes in the environmental radioactivity in human activities [2]. The basic component of our life support system is considered to be in the soil, water, plants and air. These environmental components contain measurable amount of radioactivity. The specific metabolic character of the plant species may lead to accumulation of radio-nuclides in their organs which may further depend upon the physico-chemical characteristics of the soil. Therefore, there may be increased risk to human population via food chain.

The radioactivity from the phosphate ore was not grossly affected by processing during fertilizer production. They are therefore likely to be transferred to the plants when applied to them [3]. According to the United Nations Scientific Committee on Effects of Atomic Radiation report [4], the greatest contribution to mankind’s exposure comes from natural background radiation [5]. The study of natural radioactivity is usually done in order to gain the information about the present levels of harmful pollutants discharged to the environment itself or in the living creatures. It is also important to understand the behavior of natural radionuclides in the environment, because such information can be used as the associated parameter values for the radiological assessments [6]. The soil radioactivity is usually important for the purposes of establishing baseline data for the future radiation impact assessment, radiation protection and exploration [7].

The fertilizers are essential in agriculture as they supply nutrients to the farming fields. The fertilizers used for plantation contain different elements including some natural radionuclides with their daughter decay products. Fertilizer use is a key factor for increasing agricultural production in India and its utilization has increased rapidly in the last four decades, mainly due to adoption of high yielding and expanding nutrient responsive cultivated land in large parts of the country. Phosphate rocks are the starting materials for the production of all phosphate fertilizers. Soil and fertilizers consists of naturally occurring radionuclides with their daughter decay products. Relatively large concentrations of natural radionuclides present in phosphate fertilizers contaminate the environment and agricultural lands during cultivation [8]. Fertilizers are considered as technologically enhanced natural radiation, which increase the environmental uranium and partially thorium concentrations in the environment [9]. The primary sources of elements from the environment to plants are: air, water and the soil [10]. The radionuclides present in the environment transfer to plants through (i) uptake from soil through roots, and (ii) direct absorption through aerial parts of the plants. Presence of radioactivity in plant organs has been reviewed by various workers [11,12]. There are two major pathways for human exposure to soil contamination: soil–plant–human (food chain pathway) and soil–human (incidental soil ingestion) pathways. Migration and accumulation of contaminants in the soil-plant system is complex, and assessment models commonly utilize a soil-plant concentration ratio, referred to as a transfer factor (TF), to estimate the transportation of radionuclides through the food chain. This ratio describes the amount of radionuclide expected to enter a plant from soil [13]. The study of natural radioactivity in plants and associated radiation exposure through the specific food materials is an important study. Plant roots are naturally associated with micro-organisms, and these associations can have direct or indirect effects on the mobility, availability and acquisition of elements by plants. There are different kinds of vegetables which may be roots, stems, leaves, fruits or seeds [14]. The radiological impact of the use of fertilizers in soil is due to the internal irradiation of the lung by the alpha particles, short lived radon-thoron progeny and the external irradiation of the body by gamma rays emitted from the radionuclides. Radon is carcinogenic to humans and responsible for main natural radiation exposure to human being [15-17].

In the present work, the estimation of alpha activity in leaves of lady’s finger plants grown using different types of fertilizers like cow dung manure (CDM), DAP (Diammonium Phosphate), NPK (nitrogen, phosphorus and potassium), single super phosphate (SSP), potash (PF), zinc sulphate (ZnSO_4_), urea (URA), and organic fertilizer (OF) in same amounts before the plantation of the seedlings, has been made and reported. Lady’s Finger with botanical name Abelmoschus esculentus (Linn) Moench, belongs to Malvaceae family. The present study is helpful not only for the assessment of radiation environment but also to assess the food safety due to the internal radiation.

## Materials and methods

### Track etch technique

**Figure 1:**
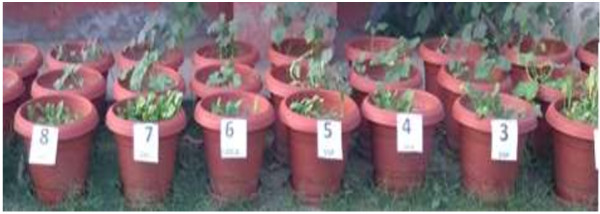
Photograph of the plants grown using different fertilizers under control study.

The tracks etch technique which is the simplest, economical, feasible and an efficient passive method has been used to determine alpha activity in plants of lady’s finger (Citrullus vul; garis Schard). The control study on lady’s finger plants was carried out during the summer season (April-June 2013) suitable for its cultivation in India. In the present control study plants (2 samples each) were grown in earthen pots having equal amounts of (12 kg) of same type of soil. Equal amounts (30 gm) of fertilizers; cow dung manure (CDM), Diammonium Phosphate (DAP), Nitrogen, phosphorus and potassium (NPK), single super phosphate (SSP), potash (PF), zinc sulphate (ZnSO_4_), urea (URA), and organic fertilizer (OF) were added to the soil during the plantation of the seeds of plants in the pots. The growth of plants in all the cases was also recorded at regular intervals of time as shown in (Figure 1). In other part of the study, the samples of complete plants were taken and dried, plastic track detectors were fixed at different parts of each plant i.e. root, stem, leaf and grain (fruit). Similar analysis was carried out after exposure time for each sample under study. The healthy leaves from different samples of plants after regular interval were plucked, dried in an oven at 40°C and then sandwiched between two plastic track detectors each with same size (2 cm × 2 cm) by wrapping a cello tape tightly to record the tracks for alpha radiations emitted from both upper and bottom faces of the leaves. The exposure time of the detectors was 60 days. At the end of exposure time, the detectors were removed and subjected to a chemical etching process in 2.5 NaOH solution at 60°C for one and half hour. The detectors were washed, dried and after that, the tracks caused by alpha radiations emitted from the leaves were counted using optical Olympus microscopes at magnification 400 X. The alpha track density from the fertilized soil was measured by placing LR-115 of size 2 × 2 cm^2^ directly over the soil in the plant pots for 7 days.

### Transfer factors

Transfer factors (TFs), which are the ratios of alpha track densities in plant leaves and soil can be used as an index for the accumulation of trace elements by plants or transfer of elements from soil to plants [18,19]. From the observed alpha track density in the plant and in the corresponding soil, the TF values were calculated according to the following equation.

(1) $$\mathrm{TF}=\frac{\mathrm{Alpha}\;\mathrm{track}\;\mathrm{density}\;\mathrm{in}\;\mathrm{leaves}\;\mathrm{of}\;\mathrm{plant}\;\left({\mathrm{Tcm}}^{-2}d^{-1}\right)}{\mathrm{Alpha}\;\mathrm{track}\;\mathrm{density}\;\mathrm{in}\;\mathrm{dry}\;\mathrm{soil}\;\left({\mathrm{Tcm}}^{-2}d^{-1}\right)}$$

### Estimation of radon exhalation rates from fertilized soil samples

The “sealed Canister technique” was used for the measurement of radon concentration and its exhalation rates in soil samples [20]. Samples of soil were collected from different earthen pots. A known amount of oven dried samples (100 gram each) were put in the plastic canister. LR-115 type-II plastic track detectors were fixed on the bottom of lid of each canister with tape such that sensitive side of the detector faced the samples. The canisters were tightly closed from the top and sealed. The size of the detectors is 2 cm × 2 cm and was exposed to samples for 100 days. The detector records the tracks of α-particles emitted by radon gas produced through α decay of radium. Radon and its daughters reach an equilibrium concentration after 4 h and hence equilibrium activity of emergent radon can be obtained from the geometry of “canister” and time of exposure. At the end of the exposure time, the detectors were removed and subjected to a chemical etching and counting process.

Mass exhalation rate ‘E_M_’ is obtained from the following formulae [21,22].

(2) $$E_{M}=\frac{\mathrm{CV\lambda}}{M\left[T+\left(1/\lambda \right)\left\{\exp\left(-\mathrm{\lambda T}\right)-1\right\}\right]}\left(\mathrm{Bq}\;{\mathrm{Kg}}^{-1}h^{-1}\right)\mathrm{for}\;\mathrm{mass}\;\mathrm{exhalation}\;\mathrm{rate}$$

Where, E_M_ is measured in Bq Kg^−1^ h^−1^; C is the Integrated radon exposure as measured by LR-115 solid state nuclear track detectors (Bq m^−3^ h^1^); M is the mass of sample (Kg); V is the volume of air in canister (m^3^); T is the time of exposure (hrs) and λ is the decay constant for radon (h^−1^). The measured track density was converted into radon concentrations using a calibration factor 0.056 tracks. cm^−2^.d^−1^/Bq.m^−3^.

## Results and discussion

### Alpha track density measured in plants

**Figure 2:**
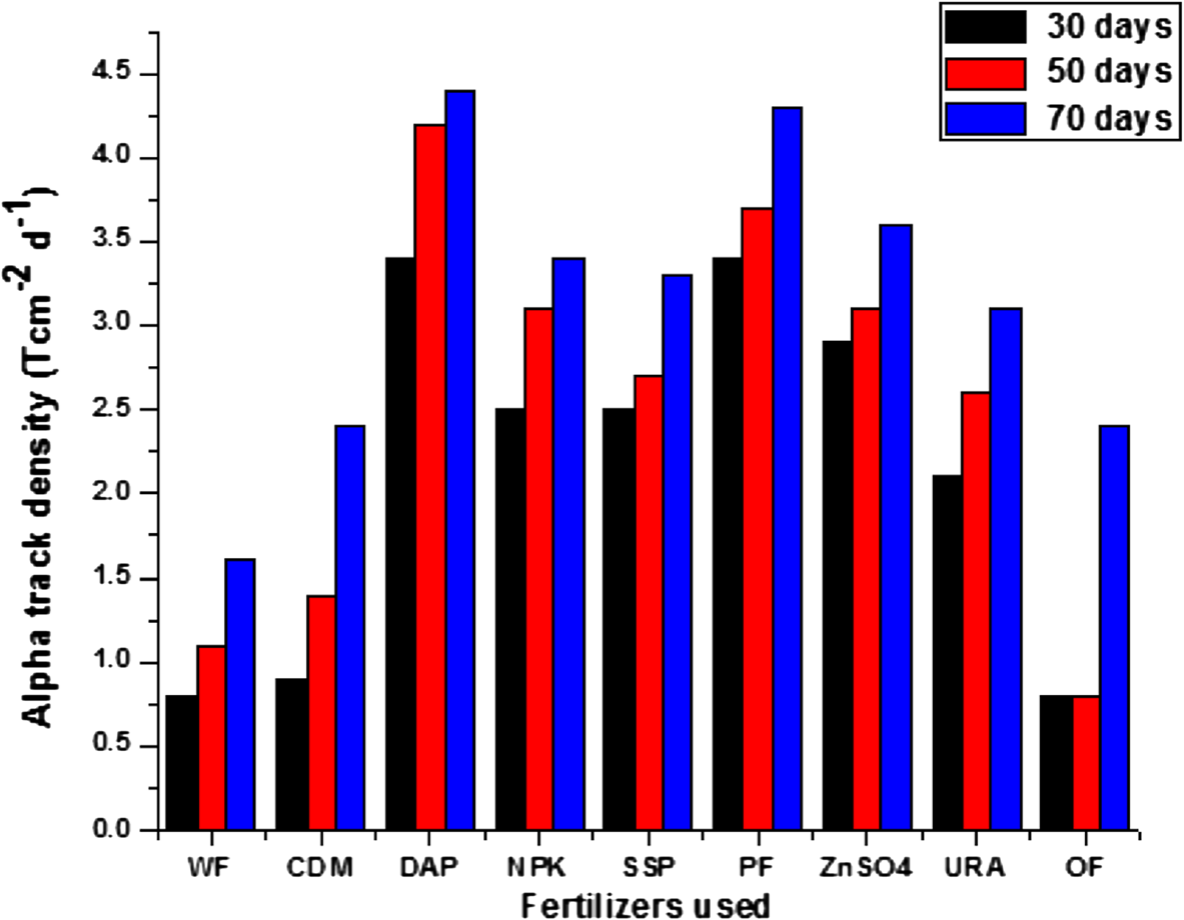
Variation in alpha track densities with different fertilizers used in Lady’s finger plants after 30, 50 and 70 days of plantation.

During control study carried out on plants grown using different fertilizers, the alpha-track density (Tcm^−2^) was measured in leaves of plants at different interval of time (Figure 2). The variation in alpha track densities was also observed in root, stem, leaf and grain parts of the plants. In case of plants grown using phosphate fertilizers the alpha radioactivity was found to be more compared with other fertilizers. For leaves plucked from the plants after 30 days of plantation of the seeds, it was found that the alpha track densities on the top face of the leaves varied from 33 to 198 T cm^−2^ while at the bottom face these varied from 38 to 248 T cm^−2^ with an average of 49 ± 11 to 206 ± 2.6 as shown in Table 1. For leaves plucked from the plants after 50 days of plantation of the seeds, it was found that the alpha track densities on the top face of the leaves varied from 33 to 232 T cm^−2^ while at the bottom face these varied from 66 to 265 T cm^−2^ with an average of 49 ± 16 to 248 ± 16 as shown in Table 2. For leaves plucked from the plants after 70 days of plantation of the seeds, it was found that the alpha track densities on the top face of the leaves varied from 83 to 232 T cm^−2^ while at the bottom face these varied from 116 to 297 T cm^−2^ with an average of 99 ± 16 to 265 ± 32 as shown in Table 3.

**Table 1:** Alpha track densities measured in the leaves of lady’s finger plants after 30 days of plantation

**Sr. no.**	**Fertilizer used**	**Tracks/cm**^**2 **^**on leaves**		AM ± SE*	**Tcm**^**−2**^**d**^**−1**^
		**Top face**	**Bottom face**		
1.	WF	33	49	49 ± 6.7	0.8
		49	66		
2.	CDM	33	66	58 ± 11	0.9
		49	83		
3.	DAP	198	215	206 ± 2.6	3.4
4.	NPK	133	165	149 ± 16	2.5
5.	SSP	133	182	149 ± 15	2.5
		116	165		
6.	PF	165	215	203 ± 18	3.4
		182	248		
7.	ZnSO_4_	149	182	174 ± 10	2.9
		165	198		
8.	URA	116	133	124 ± 11	2.1
		99	149		
9.	OF	33	38	49 ± 11	0.8
		49	83		

*AM = Arithmetic Mean, SE = Standard error (σ/ √ N), where σ is Standard deviation and N is the no. of observations.

**Table 2:** Alpha track densities measured in the leaves of lady’s finger plants after 50 days of plantation

**Sr. no.**	**Fertilizer used**	**Tracks/cm**^**2 **^**on leaves**		AM ± SE*	**Tcm**^**−2**^**d**^**−1**^
		**Top face**	**Bottom face**		
1.	WF	49	83	66 ± 17	1.1
2.	CDM	66	99	83 ± 16	1.4
3.	DAP	232	265	248 ± 16	4.2
4.	NPK	165	198	182 ± 16	3.1
5.	SSP	149	182	166 ± 16	2.7
6.	PF	215	232	224 ± 8.5	3.7
7.	ZnSO_4_	165	198	182 ± 16	3.1
8.	URA	133	182	157 ± 24	2.6
9.	OF	33	66	49 ± 16	0.8

*AM = Arithmetic Mean, SE = Standard error (σ/ √ N), where σ is Standard deviation and N is the no. of observations.

**Table 3:** Alpha track densities measured in the leaves of lady’s finger plants after 70 days of plantation

**Sr. no.**	**Fertilizer used**	**Tracks/cm**^**2 **^**on leaves**		AM ± SE*	**Tcm**^**−2**^**d**^**−1**^
		**Top face**	**Bottom face**		
1.	WF	83	116	99 ± 16	1.6
2.	CDM	133	149	141 ± 7.9	2.4
3.	DAP	232	297	265 ± 32	4.4
4.	NPK	198	215	206 ± 8.5	3.4
5.	SSP	182	215	198 ± 16	3.3
6.	PF	232	281	256 ± 24	4.3
7.	ZnSO_4_	198	232	215 ± 17	3.6
8.	URA	165	198	182 ± 16	3.1
9.	OF	49	66	57 ± 8.5	0.9

*AM = Arithmetic Mean, SE = Standard error (σ/ √ N), where σ is Standard deviation and N is the no. of observations.

**Figure 3:**
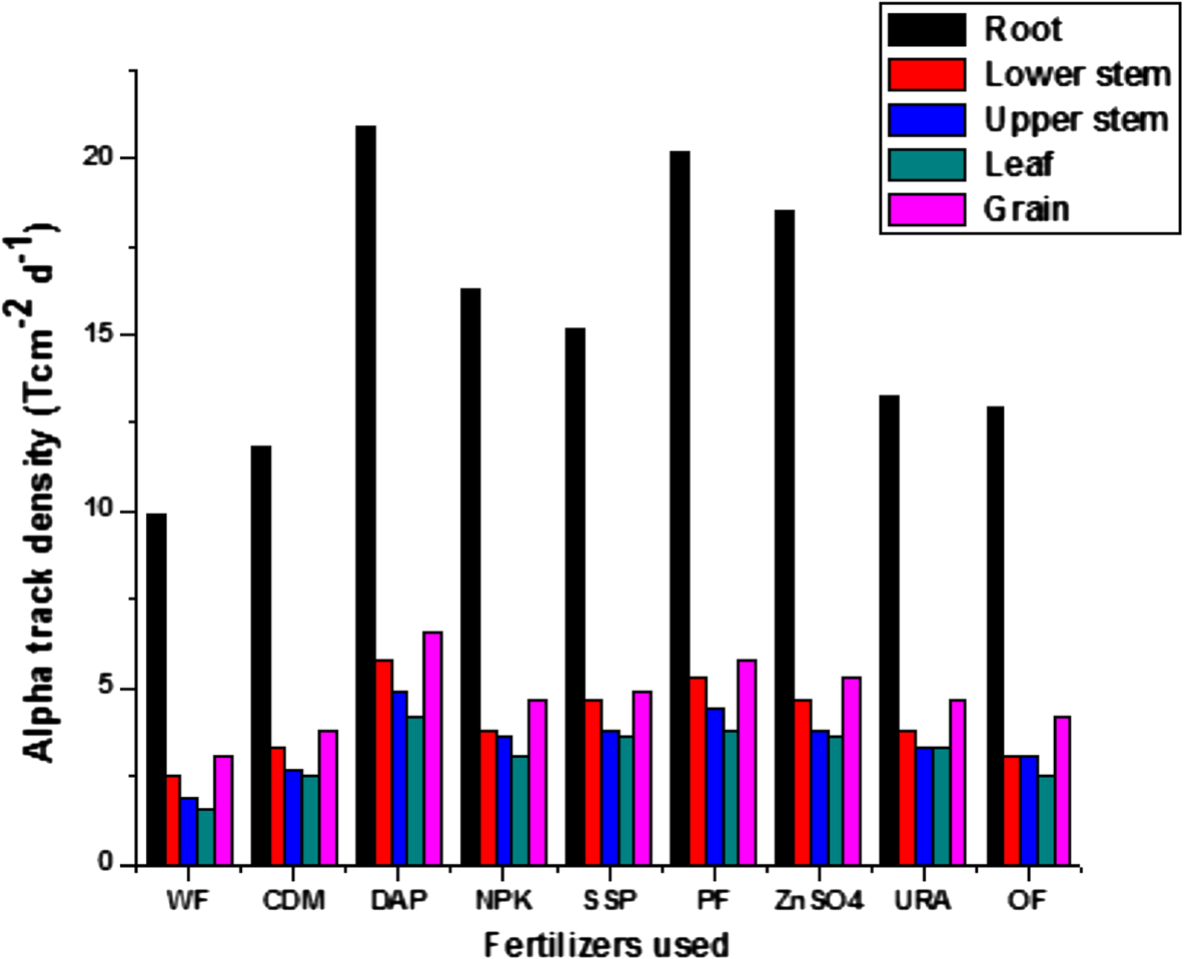
Variation in alpha track densities in roots, lower stem, upper stem, leaf and grain parts of lady’s finger plants grown using different fertilizer after 80 days of plantation.

The alpha track densities in roots, lower stems, upper stems leaves and fruit parts of lady’s finger plant after plantation varied from 9.9 to 20.6 T cm^−2^ d^−1^, 2.5 to 5.8 T cm^−2^ d^−1^, 1.9 to 4.9 T cm^−2^ d^−1^, 1.6 to 4.2 T cm^−2^ d^−1^ and 3.1 to 6.6 T cm^−2^ d^−1^ with an average of 3.8 ± 1.5 to 8.5 ± 3.2 as shown in Table 4. The alpha activity was maximum in root part of the plants and decreased upwards. There is a transportation of radioactive nuclides which may be concentrated near the roots of the plants, where casparian strip; most effective in root of plants and caused blockage of uranium in roots. The alpha track densities was found to be higher on its bottom face as compared to that on the upper face which may be due to the presence of large number of trichomes at the lower face to which dust particle in the environment with the radon daughter attached, and get stuck. For a given leaf, alpha activity to be higher in the middle portion of the leaf as compared to that in regions near the tip of leaf and the part of leaf near to the stem. This may be due to the higher glandular trichomes in the middle portion of leaves. Also the ventilation in the pheripheral part of leaf is better than that in the middle part of leaf. But it does not affect the alpha track density from leaf part as it is mostly caused by the solid radionuclide rather than radioactive gas radon. The growth observed was better in case of DAP, OF and NPK fertilizers when added before plantation and in case of URA when added few days after the plantation, compared with other fertilizers. The variation in alpha track density per day (Tcm^−2^d^−1^) for different fertilizers has been shown in (Figure 3).

**Table 4:** Alpha track densities measured in the roots, stems, leaves and fruit parts of lady’s finger plant after plantation

**Sr. no.**	**Type of fertilizer**	**Alpha track densities (Tcm**^**−2**^**d**^**−1**^**) root lower stem upper stem**			**Leaves**	**Fruit**	AM ± SE*
1.	WF	9.9	2.5	1.9	1.6	3.1	3.8 ± 1.5
2.	CDM	11.8	3.3	2.7	2.4	3.8	4.8 ± 1.7
3.	DAP	20.9	5.8	4.9	4.4	6.6	8.5 ± 3.2
4.	NPK	16.3	3.8	3.6	3.4	4.7	6.3 ± 2.5
5.	SSP	15.2	4.7	3.8	3.3	4.9	6.5 ± 2.2
6.	PF	20.2	5.3	4.4	4.3	5.8	7.9 ± 3.1
7.	ZnSO_4_	18.5	4.7	3.8	3.6	5.3	7.2 ± 2.8
8.	URA	13.3	3.8	3.3	3.1	4.7	5.7 ± 1.9
9.	OF	12.9	3.1	3.1	0.9	4.2	5.2 ± 1.9

*AM = Arithmetic Mean, SE = Standard error (σ/ √ N), where σ is Standard deviation and N is the no. of observations.

### Soil-to-plant transfer factors

**Figure 4:**
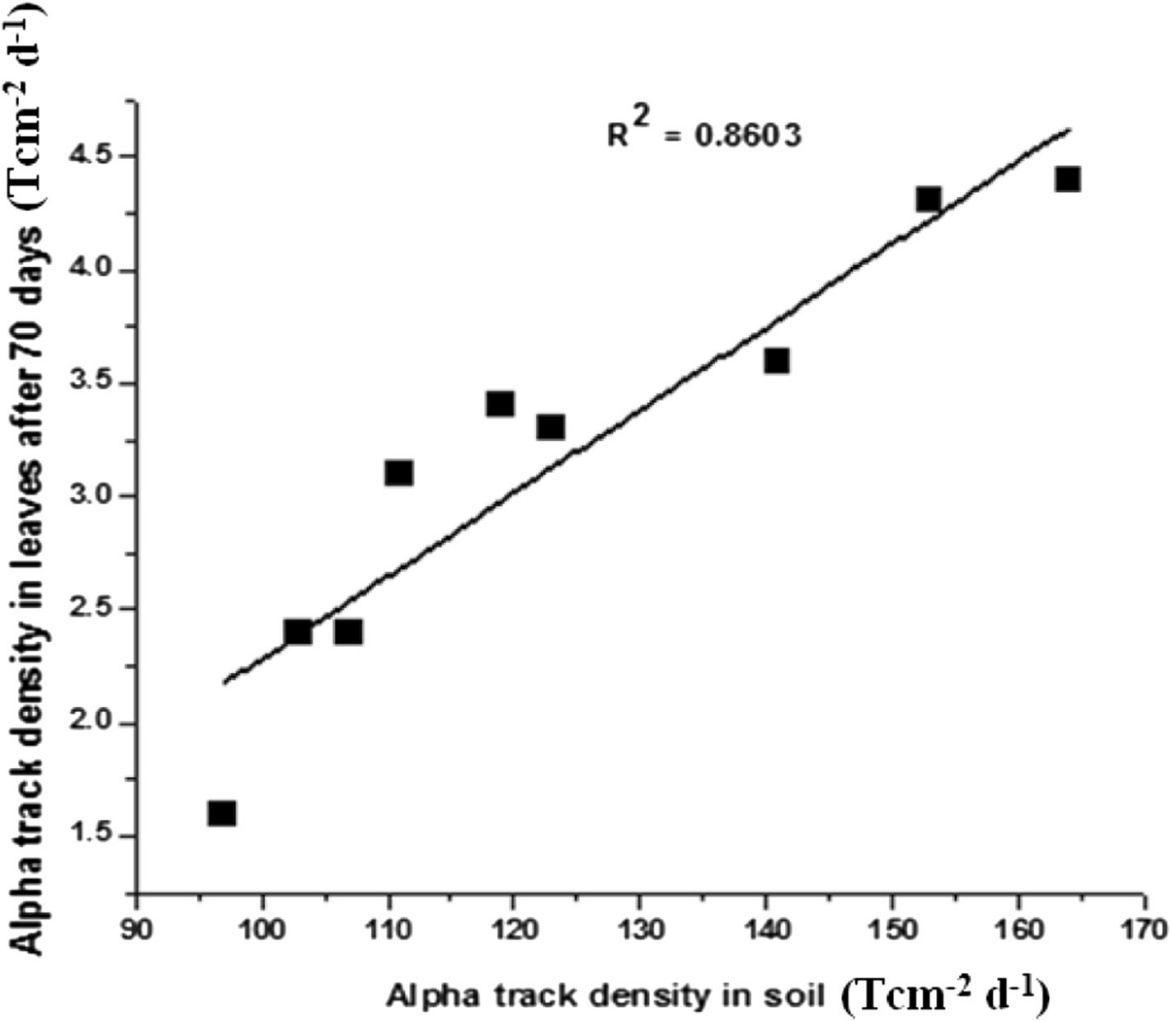
Correlation between alpha track densities for soil and plant leaves after 70 days.

Soil samples were collected from different pots and alpha activity was measured using LR- 115 solid state nuclear track detector. Using the results of alpha track densities in soil and plants the soil-to-plant transfer factors were calculated for different samples using Eq. (1). Transfer factor for 30 days samples was found in the range (0.7-2.3) × 10^−2^ while for 50 days samples range was (0.7-2.6) × 10^−2^ (Table 5). Similarly the transfer factors for 70 days samples varied in the range (1.6-2.8) × 10^−2^ respectively. The alpha track densities in soil varied from 97 Tcm^−2^d^−1^to 164 Tcm^−2^d^−1^. Using the results of alpha track densities in soil and plants, the soil-to-plant transfer factors were calculated for different leaf samples of the plants. The soil-to-plant transfer factor was also found to increase with addition of some phosphate fertilizers to soil. A positive correlation was observed between alpha track density of soil and plant samples (Figure 4).

**Table 5:** Transfer factors for different growing period of leaves of lady’s finger plant

**Fertilizer used**	**Transfer factors after**			**(Tcm**^**−2 **^**d**^**−1**^**) In soil**
	**30 days (×10**^**−2**^**)**	**50 days (×10**^**−2**^**)**	**70 days (×10**^**−2**^**)**	
WF	0.8	1.2	1.6	97
CDM	0.8	1.4	2.4	103
DAP	2.1	2.6	2.7	164
NPK	2.1	2.6	2.8	119
SSP	2.1	2.2	2.7	123
PF	2.3	2.4	2.8	153
ZnSO_4_	2.1	2.2	2.6	141
URA	1.9	2.4	2.8	111
OF	0.7	0.7	2.3	107
Range	(0.7-2.3)	(0.7-2.6)	(1.6-2.8)	97-164

### Radon concentration and exhalation rate measurements

**Figure 5:**
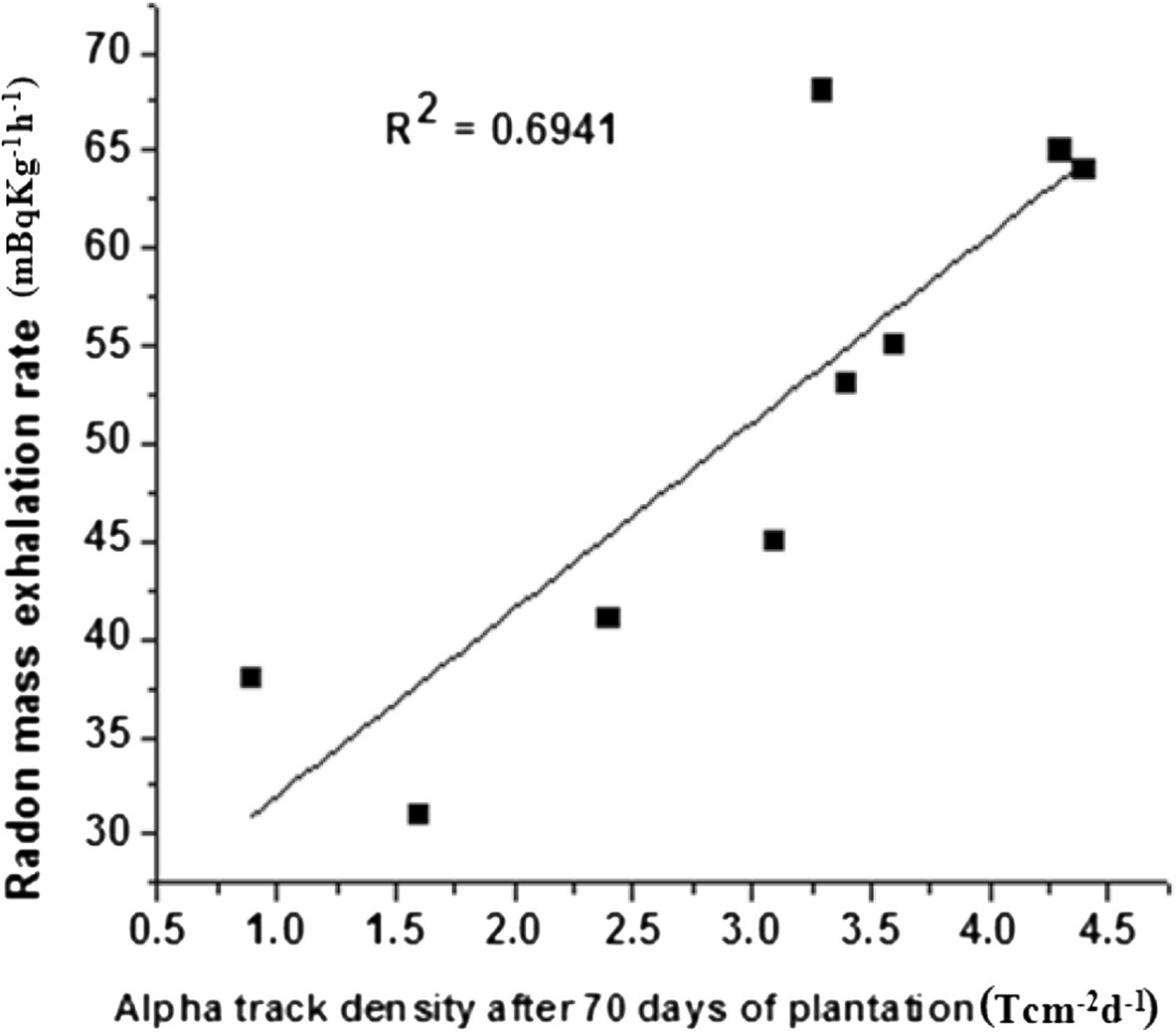
Correlation between alpha track densities in lady’s finger plants after 70 days of their plantation and radon mass exhalation rate in fertilized soil samples.

The measured radon concentration and the calculated radon mass exhalation values using Eq. (2) for various samples are presented in Table 6. The average radon concentration in various samples varied from 998 ± 62 to 2198 ± 42 Bqm^−3^ while the mass exhalation rates of radon was found to change from 31 ± 1.9 to 68 ± 1.4 mBqkg^−1^ h^−1^. A positive correlation was observed between the alpha track densities and radon mass exhalation rates with correlation coefficient (0.7864) as shown in (Figure 5). It can be seen from the results that the radon concentration varied appreciably from sample to sample but was found to satisfy the safety criteria except in phosphate fertilizers from radiation safety point of view and hence, these samples do not pose much health hazard problems.

**Table 6:** The measurement of Radon concentration, Mass exhalation rates in some fertilized soil samples used for the present study

**Sr. no.**	**Fertilizer used soil**	**Radon concentration (Bqm**^**−3**^**)**	**Radon mass exhalation rate (mBqKg**^**−1**^ **h**^**−1**^**)**
1.	WF-Soil	998 ± 62	31 ± 1.9
2.	CD-Soil	1315 ± 49	41 ± 1.5
3.	DAP-Soil	2065 ± 62	64 ± 1.9
4.	NPK-Soil	1702 ± 35	53 ± 1.1
5.	SSP-Soil	2198 ± 42	68 ± 1.4
6.	PF-Soil	2088 ± 46	65 ± 1.4
7.	ZnSO_4_-Soil	1782 ± 46	55 ± 1.4
8.	URA-Soil	1436 ± 35	45 ± 1.1
9.	OF-Soil	1249 ± 46	38 ± 1.4

Mass of the sample (M) = 0.1 Kg.

Average volume of air in the can above the sample (V) = 346.18x 10^−6^ m^3^.

## Conclusions

From present investigation we can conclude that:

•The alpha track densities vary with the nature of fertilizers added to the soil. This may be due to the variation of level in radio nuclides in the fertilizers used for the growth of plants. Transfer factor describes the amount of radionuclides expected to enter a plant from soil.

•The variation in alpha radioactivity with the passage of time of growth due to large absorption of fertilizers by plants. The alpha activity was found to be more in case of potash, D.A.P. fertilizer. This may be due to the fact that the rock mineral apatite, from which phosphate is derived, is rich in uranium and its decay products. The alpha track density was found to be higher on the bottom face as compared to that on the upper face which may be due to the presence of large number of trichomes at the lower face to which dust particle from environment with the radon daughter attached, get stuck.

## Competing interests

The authors declare that they have no competing interests.

## Authors’ contribution

The plantation of lady’s figures plants were the results of efforts made by both authors. The processing of plant after plantation (drying, chemical etching, counting and exhalation study) was made by corresponding author (PC) and the analysis/interpretation of results were made by co-author (RPC). Both authors have made contribution to the review/finalization of this manuscript and approved the final manuscript.
